# Toward Multi-Functional Road Surface Design with the Nanocomposite Coating of Carbon Nanotube Modified Polyurethane: Lab-Scale Experiments

**DOI:** 10.3390/nano10101905

**Published:** 2020-09-24

**Authors:** Sang-Guk Yum, Huiming Yin, Sung-Hwan Jang

**Affiliations:** 1 Department of Civil and Environmental Engineering, Hanyang University ERICA, Ansan, Gyeonggi-do 15588, Korea; sy2509@columbia.edu; 2 Department of Civil Engineering and Engineering Mechanics, Columbia University, New York, NY 10027, USA; yin@civil.columbia.edu

**Keywords:** multi-functional road coating, carbon nanotubes, electrical conductivity, hydrophobicity, freezing temperature sensing, Joule heating

## Abstract

A novel multi-functional road surface system is designed to improve safety, the efficiency of traffic flow, and environmental sustainability for future transportation systems. The surface coating, preforming temperature detection with heating element and hydrophobic features, were fabricated with a nanocomposite consisting of carbon nanotube (CNT) modified polyurethane (PU). The CNT/PU coating showed higher electrical conductivity as well as enhanced hydrophobic properties as the CNT concentration increased. The multifunctional properties of CNT/PU coatings were investigated for use in freezing temperature sensing and heating. The CNT/PU coatings showed high temperature sensitivity in the freezing temperature range with a negative temperature coefficient of resistance. In addition, the CNT/PU coatings had excellent heating performance due to the Joule heating effect. Therefore, the proposed CNT/PU coatings are promising for use as multifunctional road coating materials for detection of freezing temperature and deicing by self-heating.

## 1. Introduction

Improving mobility and safety is one of the biggest challenges in future transportation systems. In particular, in the winter season, snow and ice significantly impact the daily lives of the public, especially commuters. To address this challenge, many researchers are looking for smart infrastructure solutions to reduce drivers’ concerns as well as optimizing the use of the public transportation system. For example, a Road Weather Information System (RWIS) has been proposed to provide useful data such as temperature, surface state, chemical concentration, and depression of freezing points from locations around the road network. Solid and liquid chemicals such as sodium chloride, calcium chloride, potassium acetate, and calcium magnesium acetate are applied into the transport roads in order to melt ice and snow by lowering the freezing point of snow–salt mixtures. However, the disadvantages of chemicals include negative environmental impacts, corrosion of infrastructure as well as vehicles, and consumption of other resources such as salt storage and gritting trucks and manpower [1,2,3].

Multifunctional composite materials are a promising solution to improve our road system [4,5]. In order to obtain multi-functionality, several functional nanomaterials have been introduced into the polymer-based matrix. Carbon nanotubes (CNTs) have been widely used as one of the functional fillers and the capabilities of CNT reinforced composite materials have already been demonstrated. For example, Jang and Yin [6,7] fabricated highly sensitive strain and fracture sensors by dispersing carbon nanotubes as well as ferromagnetic particles in polydimethylsiloxane (PDMS). In addition to sensing applications, other capabilities have been reported for a decade. For instance, Jang and Park [8] proposed carbon nanotube reinforced composite materials for dual functions such as temperature sensing and de-icing.

In this study, we first proposed multi-functional road coating materials consisting of carbon nanotubes (CNTs) and a polyurethane (PU) matrix, widely used materials for road marking, for future transportation systems. The CNT/PU coating materials were prepared by a solution casting method for high electrical conductivity that is essential for their multi-functionality. Then, we investigated hydrophobicity and electrical conductivity for CNT/PU coatings as a function of CNT concentrations. Moreover, we evaluated the temperature as well as heating response for CNT/PU coating materials for the smart road system with minimized winter maintenance.

## 2. Materials and Methods 

Multi-walled carbon nanotubes (Industrial graded MWCNTs, 10–20 nm diameter, 20–30 μm length) were obtained from Nanolab (Waltham, MA, USA). Polyurethane (PU) was purchased from EasyComposites (Staffordshire, UK). High purity acetone (>95%) was obtained from Acros Organics Ltd. (Loughborough, UK).

The fabrication of the CNT/PU coatings followed previous studies [9,10]. First, different concentrations of CNTs were mixed in 50 mL of high purity acetone. The suspension was sonicated using a tip sonicator (Q500, QSonica, Newtown, CT, USA) for 1 h on a pulse mode (15 s on/15 s off), leading to a total energy of 10,000 J. Then, part A of the PU was mixed with the suspension and then sonicated again for 1 h. After full evaporation of the acetone, the part B was mixed with the mixture, poured into the mold and cured for 24 h.

Measurement of water contact angles was performed using a contact angle goniometer (Ossila, UK) to investigate the wetting characteristics of the CNT/PU coatings. The resistance of CNT/PU coatings was measured by digital multimeter (Keithley 2500, Beaverton, OR, USA). High purity silver paint was applied to both ends of the samples to minimize contact resistance between the sample and tip probe. The electrical conductivity of the samples was calculated by

(1) σ=L/(AR)

where *R* is the measured resistance and *L* and *A* are the length and area of samples, respectively. For temperature sensing, the change in resistance of CNT/PU coatings under freezing temperature was monitored by a digital multimeter with a data acquisition system (DAQ). The samples were placed into the environmental chamber to control temperature and humidity. The resistance for multiple CNT/PU coatings was simultaneously recorded by the multimeter. The Joule heating was applied to the CNT/PU coatings (30 × 30 × 2 mm^3^) by inducing various voltages using a bench DC power supply (Keithley 2260B, Beaverton, OR, USA). Thermal images of the samples were captured using a thermal infrared camera (TH7102WX, NEC, Tokyo, Japan).

## 3. Results

**Figure 1:**
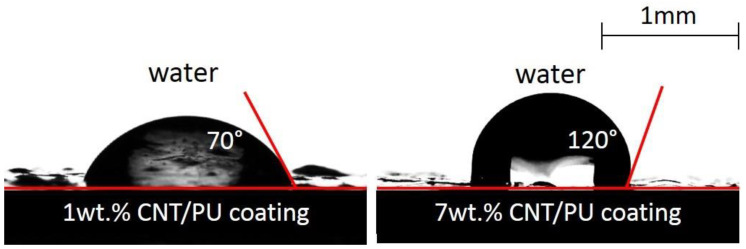
Water contact angle of CNT/PU coatings with 1 wt.% (**left**) and 7 wt.% (**right**) CNTs.

**Figure 2:**
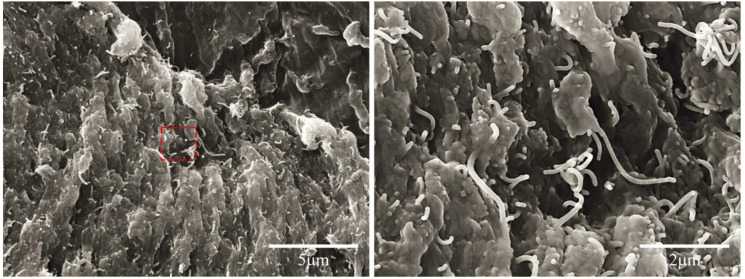
SEM images of 7 wt.% carbon nanotube modified polyurethane (CNT/PU) coatings (**left**) and enlargement of the red section (**right**).

Wettability is one of the important parameters for transport road systems. Figure 1 shows the contact angle of CNT/PU coatings. It was shown that the contact angle of the CNT/PU coating increases as the CNT concentration increases. For example, the contact angle of a 1.0 wt.% CNT/PU coating was 70° while the contact angle of a 7.0 wt.% CNT/PU coating was 120°. A higher contact angle of water for a CNT/PU coating indicates a hydrophobic transport road coating due to a microscopically thin layer of CNTs, rendering them water-repellent. The increase in contact angle for CNT/PU coatings was attributed to denser CNT networks, creating an enhanced superhydrophobic effect, as shown in Figure 2 [11].

**Figure 3:**
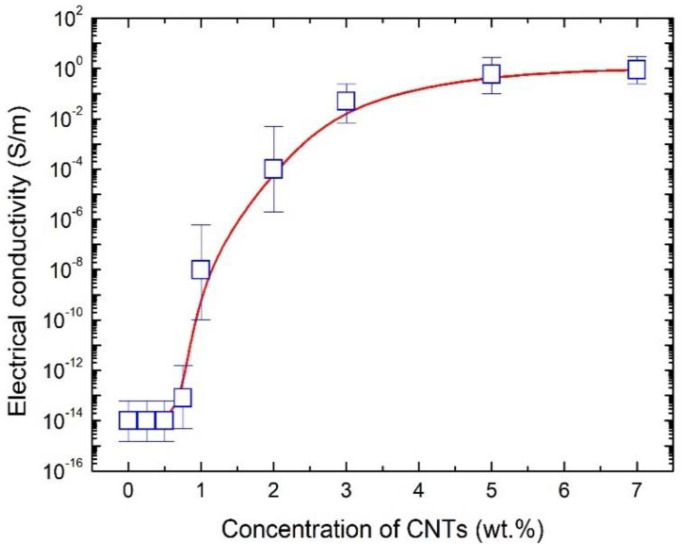
Electrical conductivity of CNT/PU coatings as a function of CNT concentrations.

Figure 3 shows the electrical conductivity of CNT/PU coatings as a function of CNT concentrations. Pristine PU presented as a non-conductive material, but the electrical conductivity of CNT/PU coatings significantly increased with increasing CNT concentration. The sharp increase in conductivity was observed between 0.75 and 1.0 wt.%, where the electrical conductivity changed from 1.5 × 10^−12^ to 6.0 × 10^−7^ S/m. This behavior has been attributed to the occurrence of a percolation transition [12,13,14]. At the percolation threshold, some CNTs begin to contact each other and a conductive path is formed, followed by a rapidly increasing number of conductive paths, as shown in Figure 2, resulting in higher electrical conductivity. In this study, we investigated the heating performance for 1.0 wt.% and 7.0 wt.% CNT/PU coatings, respectively, because both samples have different electrical conductivities. Note that the CNT/PU coatings above 7.0 wt.% showed the small increases in electrical conductivity and a high increase in viscosity.

**Figure 4:**
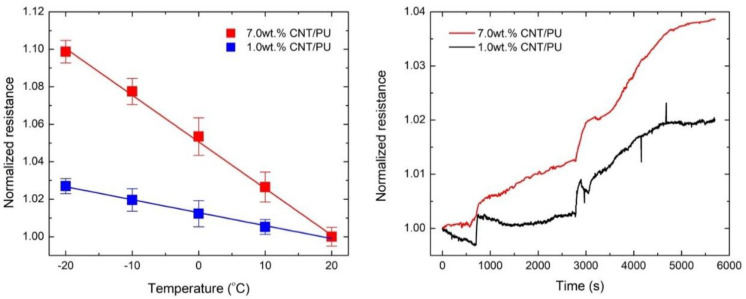
Freezing temperature sensing of the CNT/PU coatings: Normalized resistance vs. Temperature (**left**) and Normalized resistance vs. Time (**right**).

Figure 4 shows the resistance change of CNT/PU coatings at various temperatures. The resistance of the developed CNT/PU coatings increased with decreasing temperature, showing a negative temperature coefficient (NTC). The NTC effect is caused by the formation of a flocculated conductive structure under different temperatures [15]. Moreover, a higher resistance increment was observed in CNT/PU coatings with higher CNT concentrations. Figure 4 shows a real-time resistance profile of CNT/PU coatings under freezing temperatures (0 to −20 °C). The CNT/PU coating with a higher CNT concentration showed higher temperature sensitivity with less noise compared to that with the lower CNT concentration. Current temperature sensing devices are generally installed above the transport roads, leading to an inaccurate temperature. The proposed CNT/PU coating can be directly applied to the transport roads so that it can provide a more accurate temperature to users.

**Figure 5:**
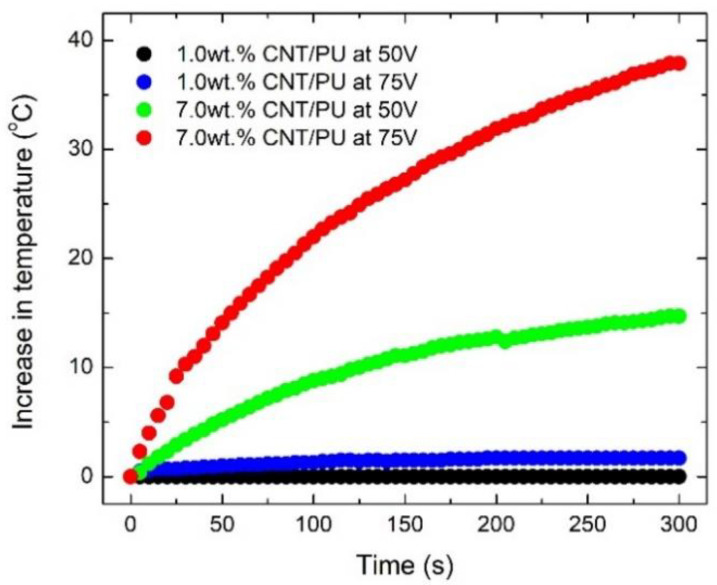
Heat profile of CNT/PU coatings due to the Joule heating effect.

**Figure 6:**
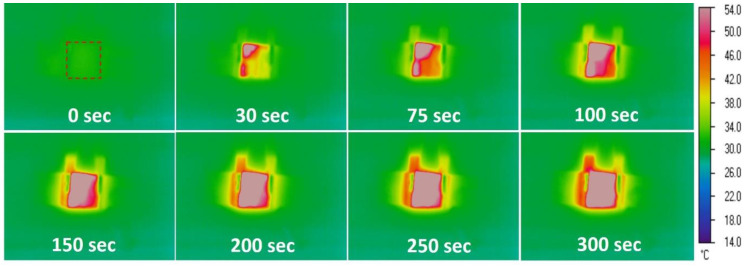
Heat evolution of a 7.0 wt.% CNT/PU coating at 75 V.

**Figure 7:**
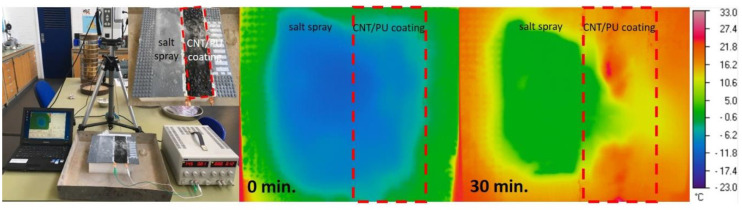
Example of Joule heating application.

**Figure 8:**
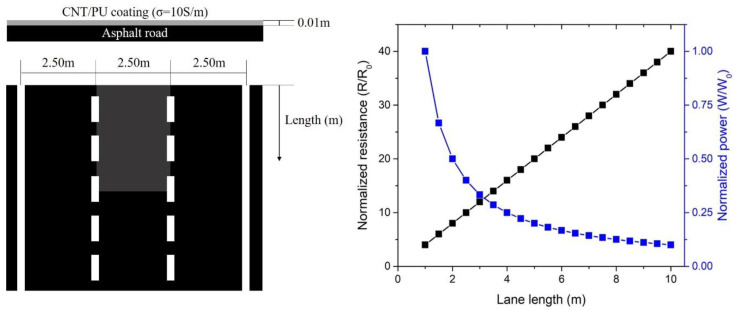
Schematic illustration of CNT/PU coating on the asphalt roads (**left**) and Estimated normalized resistance and power of the CNT/PU coating on asphalt road (**right**).

The developed CNT/PU coatings can generate a large amount of heat due to the Joule heating effect [16,17,18]. According to Joule heating, the power increased according to

(2) $$P=IV=V^{2}/R$$

where *V* is the applied voltage, *I* is the applied current, and *R* is the resistance of the CNT/PU coating. Figure 5 presents the temperature evolution for CNT/PU coatings at different applied voltages at room temperature. It was clearly shown that CNT/PU coatings with higher CNT concentrations produced faster and higher heat evolution. This is because the CNT/PU coating with a higher CNT concentration is highly conductive so that it is much more efficient to increase the temperature with a small amount of applied power. Figure 6 shows the heat evolution for a 7.0 wt.% CNT/PU coating of 30 × 30 × 2 mm^3^ at 75 V at a room temperature. It was observed that the sample provides heat quickly and uniformly, which could be a better solution for winter maintenance compared to traditional slat spraying where the melting process can take considerable time. Figure 7 demonstrates the de-icing performance of the CNT/PU coating. We sprayed water on the surface and froze it in an environmental chamber at −20 °C for 24 h. A total of 30 g of salt was sprayed onto one lane and 7 wt.% CNT/PU composites was coated onto another lane. Then, we applied a certain voltage (75 V) to the CNT/PU coating and monitored the temperature changes using an infrared thermal camera. All ice covered on the CNT/PU coating was completely melted within 30 min because of the Joule heating effect. Finally, Figure 8 provides an estimated normalized resistance and power for CNT/PU coatings on an asphalt road. It is assumed that a width of one lane is 2.5 m it is clearly shown that the normalized resistance of the CNT/PU coatings linearly increased as the length of the lane increased. Thus, the normalized power of the CNT/PU coatings decreased as the length of the lane decreased. In the future, the power would be enhanced by shortening the length of the lane and applying different electrode connections such as parallel or series connections, and increasing the electrical conductivity of CNT/PU coatings.

## 4. Conclusions

This study shows the feasibility of multi-functional CNT/PU coatings for future road surface design. Highly electrically conductive road coating materials were successfully fabricated by the dispersion of CNTs in the polyurethane matrix. The addition of CNTs showed enhanced hydrophobicity and electrical conductivity. Proposed CNT/PU coatings also provided freezing temperature sensing due to a negative temperature coefficient. In addition to the freezing temperature sensing, the CNT/PU coatings generated an amount of heat over the surface due to the Joule heating effect. In this study, the CNT/PU coating with a higher CNT concentration was more efficient to monitor the temperature and to generate heat. Therefore, we believe that highly conductive mixes consisting of CNTs and PU may have potential in smart transport road coating systems by being able to detect freezing temperatures and de-icing for winter maintenance.
